# Study of 27 Aqueous Humor Cytokines in Type 2 Diabetic Patients with or without Macular Edema

**DOI:** 10.1371/journal.pone.0125329

**Published:** 2015-04-29

**Authors:** Ning Dong, Bing Xu, Liqun Chu, Xin Tang

**Affiliations:** 1 Department of Ophthalmology, Beijing Shijitan Hospital, Capital Medical University, Beijing, People’s Republic of China; 2 Clinical College of Ophthalmology, Tianjin Medical University, Tianjin Eye Hospital, Tianjin, People’s Republic of China; Medical University of South Carolina, UNITED STATES

## Abstract

The aim of the present study was to compare the changes in the levels of 27 aqueous humor cytokines between diabetic patients with macular edema (ME) and diabetic patients without ME. Undiluted aqueous humor samples were obtained from 68 consecutive type 2 diabetic patients without ME and 56 consecutive type 2 diabetic patients with ME. The concentrations of 27 cytokines in the aqueous humor samples were measured using a multiplex bead immunoassay. Compared with diabetic patients without ME, diabetic patients with ME had significantly higher concentrations of IL-1β, IL-6, IL-8, IP-10, MCP-1, and VEGF in the aqueous humor. However, the concentrations of IL-10 and IL-12 were significantly lower in the diabetic patients with ME. The aqueous humor levels of IL-1β, IL-6, IL-8, MCP-1, IP-10, and VEGF were closely correlated with retinal macular thickness, retinal macular volume and the severity of ME. In addition, the aqueous humor levels of IL-10 and IL-12 decreased with increasing the severity of ME. A variety of cytokines associated with inflammation and angiogenesis may contribute to the pathogenesis of diabetic macular edema, and both anti-inflammatory and antiangiogenic agents should be included in the treatment of ME simultaneously.

## Introduction

Once considered a disease in the working population of the industrially developed world, diabetes mellitus (DM) has been a global health problem, with Asia accounting for 60% of the world’s diabetic population [1, 2]. Diabetic retinopathy (DR) is one of the most significant complications of DM, and it occurs in 90% of patients after 20–30 years from the disease diagnosis [3]. DR is a progressive condition with microvascular alterations that lead to retinal ischemia, retinal permeability, retinal neovascularization and diabetic macular edema (DME) [4]. DME is the leading cause of decreased visual acuity in patients with diabetic retinopathy, which affects approximately 6.8%~14% of the diabetic population [5–7].

DME is usually the leakage of intraretinal fluid from perifoveal abnormal retinal capillaries or microaneurysms, is characterized by intraretinal and subretinal accumulations of fluid. Although the pathogenesis of DME is likely multifactorial and remains unknown, it appears to be associated with breakdown of the blood–retinal barrier (BRB) and the blood–aqueous barrier (BAB). Currently, it is known that the breakdown of the vascular barrier and inflammatory processes may play a role in the development of DR, and our previous studies have demonstrated that elevated levels of angiogenic factor, inflammatory cytokines, chemokines and growth factors can be detected in the aqueous humor of patients with DR [8]. In addition, our previous study suggested that interleukin-1β (IL-1β), IL-6, monocyte chemotactic protein-1 (MCP-1), vascular endothelial growth factor (VEGF) and IL-10 may be potential predictors of postoperative macular thickness in non-diabetic patients following uncomplicated phacoemulsification cataract surgery [9]. Thus, it has been hypothesized that cytokines in the ocular fluid may be involved in the pathogenesis of diabetic macular edema. A previous study measured the concentrations of VEGF and IL-6 in aqueous humor in diabetic patients with or without macular edema by enzyme linked immunosorbent assay (ELISA) and demonstrated that high VEGF and IL-6 levels in the aqueous humor are involved in the pathogenesis of macular edema [10].

However, the limitations of the previous study on aqueous humor cytokines were the examination of a limited number of cytokines. Exploring a greater number of cytokines would provide broader insight into the inflammatory mechanisms involved. Recently, multiplex bead immunoassay has been used to detect cytokines in the aqueous humor and it can simultaneously quantify multiple cytokines in very small sample volumes [8, 9, 11–13]. Jonas JB suggested that numerous cytokines are associated with the presence of diabetic macular edema using a Luminex xMAP suspension array technology [11]. However, the number of patients enrolled in that study was relatively small [11]. In addition, their conclusions were based on comparing the levels of cytokines in diabetic patients with DME to that of non-diabetic patients [11]. So, their results not only showed the difference between diabetic macular edema and without macular edema, but also there were a confounding difference between diabetic state and non-diabetic state [11]. These researches did not completely and exactly explain why the severities of DR in diabetic patients are similar, but some patients with macular edema and others without macular edema.

In this study, therefore, we used a multiplex bead immunoassay to compare the changes in the concentrations of 27 aqueous humor cytokines in the similar severities of diabetic retinopathy patients with or without macular edema and also investigated the relationship between the levels of these factors and the severity of macular edema.

## Materials and Methods

### Subjects

Undiluted aqueous humor samples were obtained from 124 consecutive type 2 diabetic patients (124 eyes; 63 males and 61 females). 68 consecutive type 2 diabetic patients (68 eyes; 37 males and 31 females) without macular edema were undergoing cataract surgery and 56 consecutive type 2 diabetic patients (56 eyes; 26 males and 30 females) with macular edema were undergoing intravitreal injection of triamcinolone acetonide (TA) from April 2011 to June 2013. The inclusion criteria for both groups were the absence of any retinal or optic nerve disease except diffuse retinopathy. The exclusion criteria included (1) any other ocular condition (e.g., glaucoma, uveitis), (2) a history of ocular surgery, (3) a history of the intravitreal injection of triamcinolone or anti-VEGF therapy, (4) the study eyes have undergone laser therapy, and (5) a history of ocular inflammation.

This study was approved by the Ethics Committee of Beijing Shijitan Hospital, Capital Medical University, Beijing, People’s Republic of China and was performed in accordance with the Declaration of Helsinki. Written informed consent was obtained from all patients prior to their participation in the study.

### Design and Procedure

This study was a comparative cross-sectional study and was performed at Beijing Shijitan Hospital, Capital Medical University, Beijing, People’s Republic of China.

Patients underwent a complete ophthalmologic examination and a general physical examination that included assessments of visual acuity, slit lamp–assisted biomicroscopy of the anterior segment, a fundus examination, fluorescence fundus angiography (FFA), which was used for the clinical diagnosis of DR, and optical coherence tomography (OCT), which was used to measure the foveal center point thickness (FCPT). The OCT examination (Stratus OCT3; Carl Zeiss Meditec, Dublin, California, USA) was performed by an experienced operator through a dilated pupil. Each study eye underwent OCT testing fewer than 2 weeks before treatment. OCT images were generated with the use of six radial-line scans, 6.00 mm each in length. The circular map was subdivided into nine quadrants according to the Early Treatment Diabetic Retinopathy Study (ETDRS) Group and the total macular volume was determined as the sum of the volume of the nine quadrants [14]. The maximal foveal center point thickness (in micrometers) was measured at the center point of the fovea by manually placing computerized calipers at the vitreous–retina and retina–retinal pigment epithelium interfaces [15, 16]. The diabetic patients with macular edema was defined as a maximal foveal center point thickness of at least 250 μm (Stratus OCT3 value).

The severity of diabetic macular edema was confirmed by two experienced observers who were blinded as follows: no macular edema (NME), focal macular edema (FME), diffuse macular edema (DME) and cystoid macular edema (CME) [17]. FME was characterized by areas of focal fluorescein leakage from specific capillary lesions (microaneurysms and dilated capillary segments) and by retinal thickening that occupied less than 1 disc area. DME was defined as leakage from diffusely dilated retinal capillaries throughout the posterior pole associated with retinal thickening of one disk area or more, and CME was defined as a cystic pattern of hyperfluorescence in the outer plexiform layer within the macula.

The severity of diabetic retinopathy was confirmed by standardized fundus color photography and FFA and was graded using the modified severity scale from the ETDRS Group [18, 19]. In order to only show the difference of cytokine concentration in aqueous humor of eyes with diabetic macular edema and without macular edema, all of patients were similar severities of diabetic retinopathy.

### Aqueous humor sampling

At the time of cataract surgery or intravitreal injection of TA, a limbal paracentesis was made with a sterile tuberculin syringe. Undiluted aqueous humor samples (0.1–0.2 ml) were aspirated into a syringe. The samples were immediately frozen and stored at -80°C until analysis.

### Multiplex analysis of cytokines in aqueous humor samples

A Bio-Plex multiplex assay (Bio-Plex Human Cytokine 27-plex panel; Bio-Rad, Hercules, CA) was used to measure the concentrations of 27 human aqueous humor cytokines: interleukin-1β (IL-1β), IL-1rα, IL-2, IL-4, IL-5, IL-6, IL-7, IL-8, IL-9, IL-10, IL-12, IL-13, IL-15, IL-17, basic fibroblast growth factor (b-FGF), EOTAXIN, granulocyte colony-stimulating factor (G-CSF), granulocyte macrophage colony-stimulating factor (GMCSF), interferon-gamma (IFN-γ), interferon-induced protein-10 (IP-10 or CXCL10), monocyte chemotactic protein-1 (MCP-1 or CCL2), macrophage inflammatory protein-1α (MIP-1α or CCL3), macrophage inflammatory protein-1β (MIP-1β or CCL4), platelet-derived growth factor-BB (PDGF-BB), regulated upon activation normal T-cell expressed and secreted (RANTES), tumor necrosis factor-alpha (TNF-α), and vascular endothelial growth factor (VEGF).

Aqueous humor samples and cytokine standards were diluted 1:4 using manufacturer-supplied diluent (Bio-Plex Human Serum Diluent; Bio-Rad). In brief, cytokine standards or diluted aqueous humor samples were added to wells of a 96-well plate containing cytokine detection beads and incubated for 30 minutes, which were carried out at room temperature with the 96-well plate sealed and placed on an orbital shaker (300 rpm). After incubation, the plate was washed, secondary antibody (25μL) was added, and the plate was sealed and placed on an orbital shaker (300 rpm) for 30 minutes. Then the plate was washed, 50μl streptavidin-phycoerythrin detection reagent was added, and the plate was sealed and placed on an orbital shaker (500 rpm) for 10 minutes. Then the plate was washed with 100 uL Bio-Plex wash-buffer for 3 times. The beads were resuspended in 125 μL Bio-Plex assay buffer, and shaken for 30 seconds at 1100 rpm. The analysis procedure was conducted according to the manufacturer’s instructions. Standard curves were generated using the reference cytokine sample supplied in the kit and were generated using the Bio-PlexTM 200 System (software version 6.0; Bio-Rad Laboratories) and were used to calculate the cytokine concentrations in aqueous humor samples.

### Statistical analysis

Data were recorded as the means±SD or the median and range. The statistical analyses were performed using the program SPSS for Windows Version 17.0. The Pearson X^2^ test was used to compare the proportions of qualitative variables. Student’s t-test and the Mann-Whitney U test were used to compare the means of the quantitative variables between two independent groups. The Kruskal-Wallis test was used to compare multiple groups. Spearman’s rank-order correlation coefficients and multiple linear regression analysis were used to assess the relationship between the concentrations of the assayed cytokines and the severity of DR. A *P* value less than 0.05 was accepted as statistically significant.

## Results

### Patient demographics

A total of 124 consecutive type 2 diabetic patients (124 eyes; 63 males and 61 females) were enrolled, and there were no cases of intraoperative vitreous loss or suprachoroidal hemorrhage. Table 1 shows demographic and clinical characteristics of patients, including the 68 diabetic patients without macular edema [ME (-)] and 56 diabetic patients with macular edema [ME (+)] patients. There were no significant differences in gender, age, and the severity of diabetic retinopathy between the ME (+) and ME (-) groups, but there are significant differences in hypertension. Participants with ME (+) were had a longer duration of diabetes. In addition, the levels of blood glucose and glycosylated hemoglobin in the ME (+) patients were significantly higher.

**Table 1:** Baseline characteristics of type 2 diabetic patients with macular edema [ME (+)] and without macular edema [ME (-)].

Characteristics	ME (-)	ME (+)	*P* value
Number	68	56	-
Gender			0.376*
Male (%)	37 (54.4)	26 (46.4)	
Female (%)	31 (45.6)	30 (53.6)	
Age (SD)	65.71 (7.35)	68.92 (8.12)	0.324^†^
Duration of diabetes, y (SD)	13.2 (10.5)	16.7 (11.2)	0.015^†^
Hypertension (%)	31 (45.6)	37 (66.1)	0.023*
Blood glucose level, mmol/l (SD)	7.62 (2.18)	9.12 (3.26)	0.024^†^
Glycosylated hemoglobin (SD)	7.78 (2.23)	8.66 (2.32)	0.032^‡^
Retinal macular thickness, μm (SD)	214.41 (20.70)	431.84 (89.98)	<0.001^†^
Retinal macular volume, mm^3^ (SD)	2.34 (1.49)	7.94 (1.82)	<0.001^†^
ETDRS retinopathy severity			0.328*
level 43 (%)	28 (41.2)	21 (37.5)	
level 47 (%)	34 (50.0)	25 (44.6)	
level 53 (%)	6 (8.8)	10 (17.9)	

*Pearson χ^2^ test

^†^Student’s t-test

^‡^Mann-Whitney U test

### Cytokines concentrations in the aqueous humor

Table 2 shows the concentrations of the assayed cytokines. The positive detection rates were more than 80% for 22 cytokines. The positive detection rates for the other 5 cytokines were as follows: TNF-α (58%), IFN-γ (46%), G-CSF (38%), IL-17 (26%), and MIP-1α (16%). These 5 cytokines were not included in the statistical analysis because of the low detection rates.

**Table 2:** The concentrations of the assayed cytokines in the aqueous humor samples of type 2 diabetic patients with macular edema [ME (+)] and without macular edema [ME (-)] (pg/ml).

Cytokine	ME (-), n = 68		ME (+), n = 56		*P* *
	Median	Range	Median	Range	
IL-1β	5.0	0–75	12.0	3–104	0.003^†^
IL-1rα	11.3	2–312	17.1	5–326	0.423
IL-2	1.6	0–89	1.6	0–103	0.465
IL-4	1.3	0–106	1.5	0–123	0.778
IL-5	1.1	0–103	1.3	0–116	0.689
IL-6	17.0	3–226	55.5	12–362	<0.001^†^
IL-7	4.3	0–81	2.8	0–85	0.212
IL-8	12.5	0–102	33.5	0–198	<0.001^†^
IL-9	3.1	0–112	3.2	0–163	0.661
IL-10	6.0	0–26	4.0	0–15	0.001^†^
IL-12	13.0	0–42	8.0	0–36	0.009^†^
IL-13	2.0	0–29	1.9	0–35	0.553
IL-15	1.7	0–86	1.6	0–36	0.723
IL-17	-	-	-	-	-
b-FGF	12.3	2–155	11.8	3–152	0.525
Eotaxin	5.8	0–84	6.1	0–92	0.693
G-CSF	-	-	-	-	-
GM-CSF	9.3	0–79	9.9	0–86	0.813
IFN-γ	-	-	-	-	-
IP-10	7.0	0–56	14.0	0–86	<0.001^†^
MCP-1	204.5	58–1623	632.5	126–2388	<0.001^†^
MIP-1α	-	-	-	-	-
MIP-1β	25.8	6–166	28.5	9–168	0.697
PDGF-BB	3.2	0–39	3.1	0–41	0.588
RANTES	4.5	0–76	4.8	0–69	0.658
TNF-α	-	-	-	-	-
VEGF	405.0	43–1785	1162.5	216–2546	<0.001^†^

*Mann-Whitney U test

^†^ Statistically significant

Compared to the ME (-) group, the concentrations of IL-1β (*P* = 0.003), IL-6 (*P*<0.001), IL-8 (*P*<0.001), IP-10 (*P*<0.001), MCP-1 (*P*<0.001), and VEGF (*P*<0.001) from the ME (+) patients were significantly higher. However, the concentrations of IL-10 (*P* = 0.001) and IL-12 (*P* = 0.009) in the samples from the ME (+) patients were significantly lower than the concentrations in the ME (-) patients. There were no significant differences in other cytokine concentrations between the ME (-) and ME (+) patients.

### Associations for the concentrations of the cytokines

Table 3 shows these observed cytokines are related, except for IP-10 with VEGF (r = 0.126, *P* = 0.231) and IL-10 (r = -0.161, *P* = 0.216); IL-10 with IL-12 (r = 0.255, *P* = 0.352).

**Table 3:** Correlations for the concentrations of the cytokines.

	IL-6		IL-8		IP-10		MCP-1		VEGF		IL-10		IL-12	
Variables	r	*P* *	r	*P* *	r	*P* *	r	*P* *	r	*P* *	r	*P* *	r	*P* *
IL-1β	**0.733**	**<0.001** ^†^	**0.657**	**<0.001** ^†^	**0.465**	**0.006** ^†^	**0.863**	**<0.001** ^†^	**0.812**	**<0.001** ^†^	**-0.246**	**0.012** ^†^	**-0.302**	**0.006** ^†^
IL-6			**0.769**	**<0.001** ^†^	**0.523**	**0.003** ^†^	**0.816**	**<0.001** ^†^	**0.756**	**<0.001** ^†^	**-0.308**	**0.007** ^†^	**-0.219**	**0.009** ^†^
IL-8					**0.438**	**0.005** ^†^	**0.761**	**<0.001** ^†^	**0.735**	**<0.001** ^†^	**-0.267**	**0.016** ^†^	**-0.321**	**0.013** ^†^
IP-10							**0.423**	**0.003** ^†^	**0.126**	**0.231**	**-0.161**	**0.216**	**-0.278**	**0.011** ^†^
MCP-1									**0.821**	**<0.001** ^†^	**-0.312**	**0.006** ^†^	**-0.277**	**0.018** ^†^
VEGF											**-0.237**	**0.006** ^†^	**-0.261**	**0.012** ^†^
IL-10													**0.255**	**0.352**

*Pearson correlation coefficient

^†^Statistically significant

### Association between cytokines concentrations and retinal macular thickness

**Fig 1:**
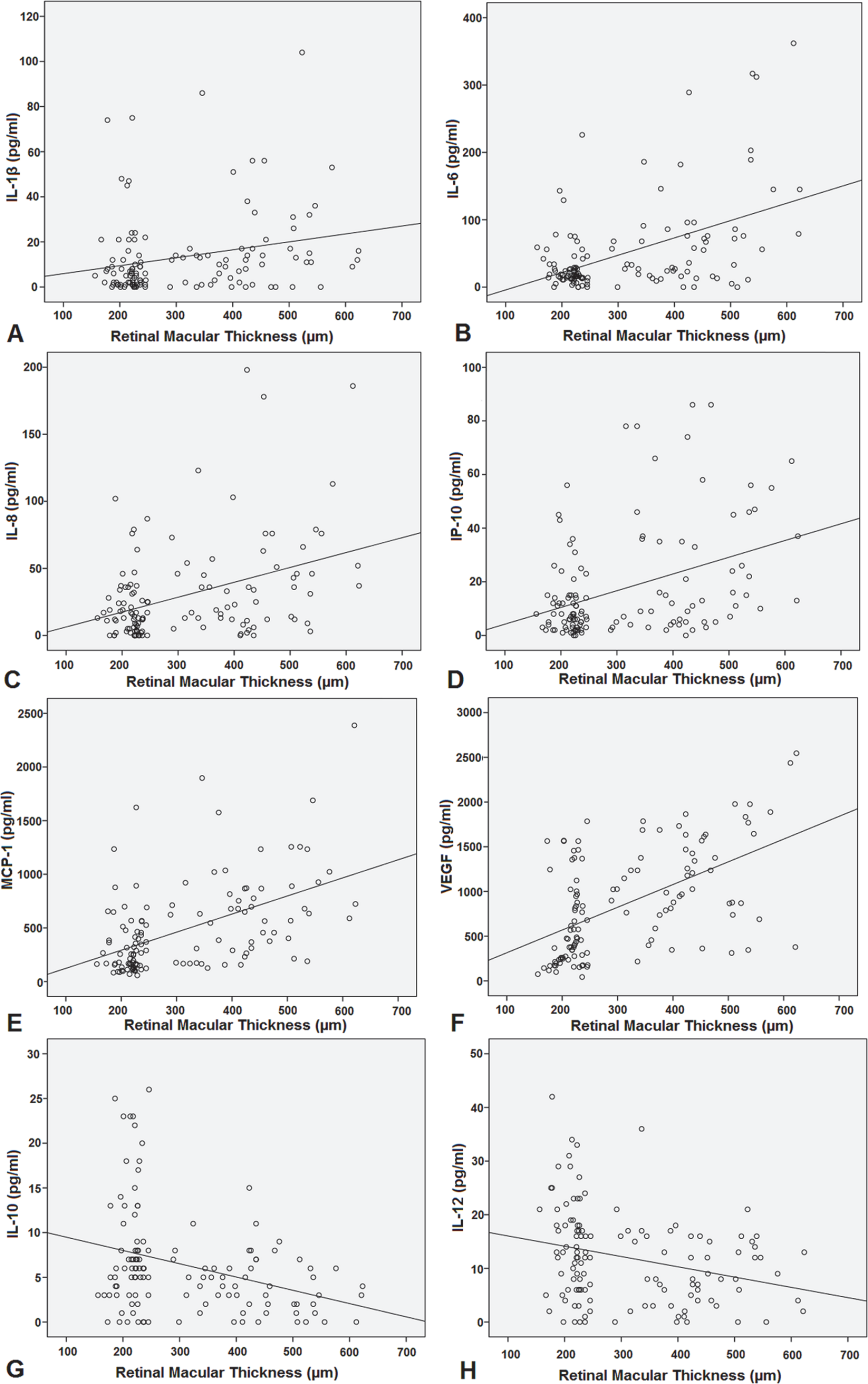
Scatterplots of the correlations between retinal macular thickness and the aqueous humour concentrations of cytokines. A. IL-1β. B. IL-6. C. IL-8. D. IP-10. E. MCP-1. F. VEGF. G. IL-10. H. IL-12.

Table 4 and Fig 1 show the relationship between the concentrations of assayed cytokines and retinal macular thickness. The aqueous levels of IL-1β (r = 0.238), IL-6 (r = 0.477), IL-8 (r = 0.382), IP-10 (r = 0.386), MCP-1 (r = 0.504), and VEGF (r = 0.543) were found to positively correlate with retinal macular thickness. In addition, the aqueous level of IL-10 (r = -0.322) and IL-12 (r = -0.277) was negatively correlated with retinal macular thickness. Furthermore, the multiple regression analysis revealed independent influences of IL-1β (β = 0.107), IL-6 (β = 0.203), IL-8 (β = 0.156), IP-10 (β = 0.165), MCP-1 (β = 0.321), VEGF (β = 0.405), IL-10 (β = -0.135), and IL-12 (β = -0.048) on retinal macular thickness.

**Table 4:** Correlations between the concentrations of the cytokines in the aqueous humor samples of type 2 diabetic patients and retinal macular thickness.

	Univariate		Multivariate	
Cytokine	r	*P* value*	β	*P* value^†^
IL-1β	0.238	0.008^‡^	0.107	0.023^‡^
IL-6	0.477	<0.001^‡^	0.203	0.011^‡^
IL-8	0.382	0.001^‡^	0.156	0.022^‡^
IP-10	0.386	<0.001^‡^	0.165	0.021^‡^
MCP-1	0.504	<0.001^‡^	0.321	<0.001^‡^
VEGF	0.543	<0.001^‡^	0.405	<0.001^‡^
IL-10	-0.322	0.001^‡^	-0.135	0.035^‡^
IL-12	-0.277	0.002^‡^	-0.100	0.048^‡^

*Pearson’s univariate correlation coefficients

^†^Multiple regression analysis

^‡^Statistically significant

### Association between cytokines concentrations and retinal macular volume

Table 5 shows the association between the concentrations of assayed cytokines and retinal macular volume. The aqueous levels of IL-1β (r = 0.254), IL-6 (r = 0.456), IL-8 (r = 0.427), IP-10 (r = 0.402), MCP-1 (r = 0.531), and VEGF (r = 0.504) were found to positively correlate with retinal macular volume. In addition, the aqueous level of IL-10 (r = -0.346) and IL-12 (r = -0.680) was negatively correlated with retinal macular volume. Furthermore, the multiple regression analysis revealed independent influences of IL-1β (β = 0.025), IL-6 (β = 0.121), IL-8 (β = 0.205), IP-10 (β = 0.172), MCP-1 (β = 0.346), VEGF (β = 0.360), IL-10 (β = -0.155), and IL-12 (β = -0.083) on retinal macular volume.

**Table 5:** Correlations between the concentrations of the cytokines in the aqueous humor samples of type 2 diabetic patients and retinal macular volume.

	Univariate		Multivariate	
Cytokine	r	*P* value*	β	*P* value^†^
IL-1β	0.254	0.004^‡^	0.025	0.012^‡^
IL-6	0.456	<0.001^‡^	0.121	0.025^‡^
IL-8	0.427	<0.001^‡^	0.205	0.002^‡^
IP-10	0.402	0.003^‡^	0.172	0.014^‡^
MCP-1	0.531	<0.001^‡^	0.346	<0.001^‡^
VEGF	0.504	<0.001^‡^	0.360	<0.001^‡^
IL-10	-0.346	0.002^‡^	-0.155	0.014^‡^
IL-12	-0.680	0.003^‡^	-0.083	0.045^‡^

*Pearson’s univariate correlation coefficients

^†^Multiple regression analysis

^‡^Statistically significant

### Association between cytokines concentrations and the severity of macular edema

**Fig 2:**
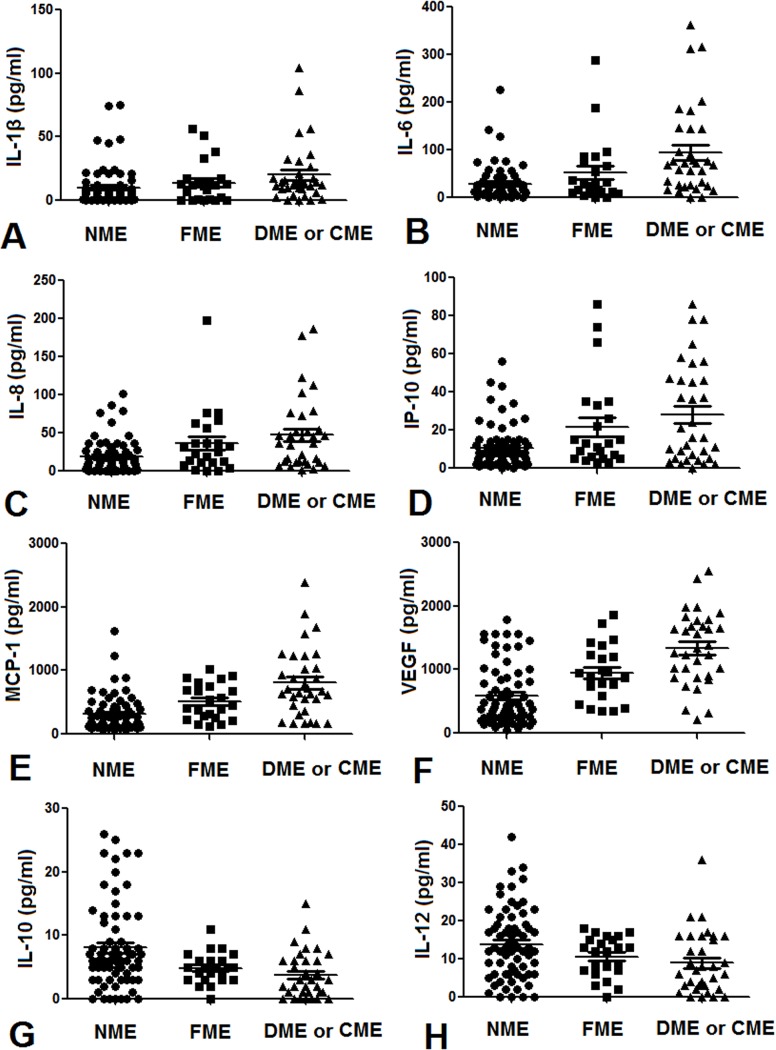
Relationship between the severity of macular edema and the aqueous humour concentrations of cytokines. A. IL-1β. B. IL-6. C. IL-8. D. IP-10. E. MCP-1. F. VEGF. G. IL-10. H. IL-12.

Table 6 and Fig 2 show the relationship between the concentrations of the assayed cytokines and the severity of macular edema. The aqueous humor levels of IL-1β, IL-6, IL-8, MCP-1, IP-10, and VEGF increased with increasing severity of macular edema. In addition, the aqueous humor levels of IL-10 and IL-12 decreased with increasing severity of macular edema, and this negative correlation was significant.

**Table 6:** Relationship between the concentrations of the assayed cytokines and the severity of macular edema.

Level	N	IL-1β (SD)	IL-6 (SD)	IL-8 (SD)	IP-10 (SD)	MCP-1 (SD)	VEGF (SD)	IL-10 (SD)	IL-12 (SD)
NME	68	9.57 (15.52)	29.22 (36.02)	19.18 (21.92)	10.81 (11.54)	314.87 (280.27)	590.31 (473.30)	8.07 (6.75)	13.89 (9.34)
FME	23	13.91 (16.03)	51.69 (67.71)	36.52 (42.59)	21.74 (23.67)	513.17 (278.69)	945.43 (437.15)	4.87 (2.40)	10.61 (5.31)
DME or CME	33	20.15 (23.67)	93.85 (93.70)	47.48 (47.39)	28.24 (26.04)	805.00 (537.44)	1337.81 (568.65)	3.75 (3.69)	8.93 (8.15)
*P* value*		0.005^†^	<0.001^†^	0.001^†^	0.002^†^	<0.001^†^	<0.001^†^	0.002^†^	0.018^†^

NME = no macular edema, FME = focal macular edema, DME = diffuse macular edema and CME = cystoid macular edema

*Kruskal-Wallis test

^†^Statistically significant

## Discussion

Diabetic macular edema is a leading cause of visual dysfunction in patients with diabetes, and the incidence increases with the duration of the diabetes, which the prevalence is 5% within the first 5 years after diagnosis and 15% at 15 years [20]. It is now widely accepted in clinical practice that diabetic macular edema can occur at any stage of DR. Epidemiology studies have shown a strong association between hypertension and DME, and intensive glycemic and blood pressure control can substantially reduce the onset and progression of DME [21, 22]. Consistent with previous studies [21, 22], our studies show the levels of blood glucose level and glycosylated hemoglobin in the ME (+) patients were significantly higher. In addition, there were significant differences in hypertension between the ME (+) and ME (-) groups. However, the pathogenesis of DME is still uncertain and requires more experience.

Undiluted aqueous humor samples were obtained from 102 nondiabetic patients and 136 consecutive diabetic patients in our previous studies, which have demonstrated that elevated levels of angiogenic factor, inflammatory cytokines and chemokines can be detected in the aqueous humor of patients with DR comparing to that of the nondiabetic controls [8]. However, it is unknown why the patients are similar severities of diabetic retinopathy, but some diabetic patients with macular edema and others without macular edema. Although landmark studies have shown that DME was associated with breakdown of the BRB and BAB and demonstrated high VEGF and IL-6 levels in the aqueous humor are involved in the pathogenesis of macular edema [10], the limitations of the previous study on aqueous humor cytokines were a limited number of cytokines. In this study, therefore, we compared the changes in the concentrations of 27 aqueous humor cytokines in the similar severities of diabetic retinopathy patients with or without macular edema. The present study showed that the aqueous humor levels of IL-1β, IL-6, IL-8, MCP-1, IP-10, and VEGF were higher in diabetic patients with macular edema and the levels of these cytokines were closely correlated with the retinal macular thickness, retinal macular volume and the severity of macular edema. In addition, the aqueous humor levels of IL-10 and IL-12 were significantly lower in diabetic patients with macular edema, and the concentrations of these two cytokines decreased with increasing severity of macular edema.

These changed cytokines, which are angiogenic and inflammatory factors, are synthesized by a variety of cells [23]. IL-8 (CXCL8) and IP-10 (CXCL10) play roles in inflammatory mechanisms of DME in the present study, which are produced by activated inflammatory cells, such as monocytes/macrophages and neutrophils [24, 25].

In addition, a number of studies have also demonstrated that intravitreal ranibizumab for anti-VEGF therapy was an effective therapeutic strategy for management of DME [26, 27]. These findings postulated that VEGF and neovascularization are an important molecular mechanism in the development and progression of diabetic macular edema. Therefore, proangiogenic factors were investigated in the present study, which are the potential component of DME. VEGF is a multifunctional cytokine, which is not only a major mediator of retinal angiogenesis, but also a potent inducer of vasopermeability [28]. Consistent with previous studies [10], levels of VEGF in aqueous humor in diabetic patients with macular edema have been found to be markedly increased.

Furthermore, IL-1β, IL-6, MCP-1 (CCL2), and IL-10 are related with both inflammation and angiogenesis simultaneously [29–36]. IL-1β, IL-6, and MCP-1 have the double function of promoting inflammation and neovascularization [29–33]. However, IL-10 is anti-inflammatory and anti-angiogenic mediator [34–36]. Our results suggest that high levels of IL-1β, IL-6, and MCP-1 (proinflammatory and proangiogenic) and low levels of IL-10 (anti-inflammatory and anti-angiogenic) are involved in the pathogenesis of diabetic macular edema.

Compared with our previous studies [8], the current results indicate that more elevated levels of angiogenic factor, inflammatory cytokines and chemokines can be detected in the aqueous humor of patients with DME comparing to that of the nondiabetic controls and the diabetic patients without macular edema. DR is a potentially sight-threatening microvascular complication of diabetes and also a low-grade subclinical inflammatory disease, which is characterized by retinal microvascular damage leading to vascular leakage and ischemia-induced retinal neovascularization. DME, which can occur at any stage of DR, is related with dilated capillaries, retinal microaneurysms, loss of pericytes, breakdown of the blood–retinal barrier (BRB), and increase in vascular permeability. Therefore, our findings may suggest that the onset of diabetic macular edema is associated with more elevated levels of angiogenic factor, inflammatory cytokines and chemokines released by macrophages, neutrophils, and endothelial cells, which are activated by four classic pathways: the polyol pathway, increased advanced glycation end-product (AGE), protein kinase C (PKC) activation, and the superoxide pathway [23].

The limitations of our study should be noted. First, the concentrations of the cytokines in vitreous samples were not determined. The cytokine levels in the vitreous are usually higher, and the analysis of vitreous would more accurately reflect the intraocular levels of cytokines and the status of the retina. However, in contrast to vitreous samples, obtaining aqueous fluid samples from the anterior chamber is easier, faster and less risky. In addition, multiplex bead immunoassay has the limitation if the cytokine levels are very low, so the positive detection rates for the 5 cytokines were not more than 80% and these cytokines were not included in the statistical analysis because of the low detection rates in the current study.

In conclusion, our study indicates that a variety of cytokines associated with inflammation and angiogenesis may contribute to the pathogenesis of diabetic macular edema and the intravitreal treatment of macular edema should be included in comprehensive DR treatment plans and that both anti-inflammatory and anti-neovascularization agents should be used simultaneously.
